# Deciphering macronutrient information about human milk

**DOI:** 10.1038/s41372-024-02029-8

**Published:** 2024-06-15

**Authors:** Mandy B. Belfort, Lisa Stellwagen, Krysten North, Sharon Unger, Deborah L. O’Connor, Maryanne T. Perrin

**Affiliations:** 1 Department of Pediatrics, Brigham and Women’s Hospital and Harvard Medical School, Boston, MA USA; 2 Department of Pediatrics, University of California, San Diego, School of Medicine, San Diego, CA USA; 3 IWK Health, and Dalhousie University, Halifax, NS Canada; 4 Department of Nutritional Sciences, University of Toronto, and the Department of Pediatrics, Sinai Health, Toronto, ON Canada; 5 Translational Medicine Program, The Hospital for Sick Children, Toronto, ON Canada; 6 Department of Nutrition, University of North Carolina Greensboro, Greensboro, NC USA

**Keywords:** Paediatrics, Nutritional supplements

## Abstract

Clinicians caring for small, vulnerable newborns increasingly have access to specific nutritional information about human milk through point-of-care analyzers and labeled products. *It is critical for clinicians to recognize that there is considerable variability in how human milk nutritional data are derived and reported*, which impacts the interpretation of nutritional values, comparison of nutritional data between products, and ultimately the ability to deliver optimal nutritional care. This article distills key issues that will enable clinicians to interpret human milk nutritional labels/analysis more effectively, ultimately allowing them to make better decisions about dietary strategies. We aim to empower clinicians to ask questions about milk sampling techniques, reported nutrient values, analysis techniques, and milk bank pooling practices. This knowledge can put human milk nutrient values in context, improve clinical care, and help to drive more rigorous research for exploring the impact of human milk feeding on infant outcomes.

## Introduction

Increasingly, clinicians caring for small vulnerable newborns have access to nutritional information about mother’s own milk (MOM) and pasteurized donor human milk (PDHM) through point-of-care analyzers and labeled products. *It is critical for clinicians to recognize that there is considerable variability in how nutritional data for human milk are derived and reported*, which impacts the interpretation of nutritional values, comparison of nutritional data between human milk products, and ultimately the ability to deliver optimal nutritional care. The purpose of this article is to distill key issues that will enable clinicians to more effectively interpret point-of-care nutritional data and thereby make better decisions about infant dietary strategies. We focus primarily on nutritional information derived using infrared spectroscopy (IR) because this technology is approved by the United States Food and Drug Administration (FDA) for use in a clinical setting and is commonly used by non-profit milk banks for labeling PDHM. IR is a technique for measuring macronutrients in milk based on the vibration patterns of different chemical bonds. This technique originated in the dairy industry; therefore, differences between the human milk matrix and the bovine milk matrix – including presence and abundance of oligosaccharides and nitrogen containing substances – must be taken into consideration when interpreting results. Where more accurate methods for determining nutritional values for human milk are available, we will also discuss these. Table 1 provides a summary of human milk macronutrients and measurement considerations.

**Table 1:** Summary of human milk macronutrient analysis and clinical implications.

Category	Analyte	Definition	Measurement notes & clinical implications
Carbohydrates	Lactose	Makes up 70–85% of the total carbohydrates in human milk. Digestible by infant for energy.	• IR carbohydrate values depend on whether instrument was calibrated to total carbohydrates (includes lactose and HMOs) or lactose. • While IR is not strongly correlated with chemical methods for measuring carbohydrates, it is accurate within FDA requirement of +/−15% • IR instruments calibrated to total carbohydrates will overestimate the available energy in human milk. • Reliable methods for measuring lactose that are not influenced by HMOs include AOAC 984.22 and AOAC 2006.06.
	HMOs	Makes up 15–25% of the total carbohydrates in human milk. Not digestible by infant. Serves multiple functions in the infant gut.	
	Monosaccharides	Makes up less than 1% of the total carbohydrates in human milk.	
	Total carbohydrates	Sum of monosaccharides, lactose, and HMOs.	
Fat	Total fat	Primarily in the form of triglycerides; a major contributor to energy and the most variable macronutrient in human milk.	• Total fat values are profoundly influenced by sample collection and handling practices. • Lack of proper sample handling may contribute to errors and bias. • IR provides reliable values for total fat, provided proper sample handling techniques are used. • Creamatocrit reliability decreases when fat concentrations are low, and when milk has undergone storage.
Protein	Crude Protein	Also referred to as Total Protein. Assumes all the nitrogen in milk is from protein; therefore, crude/total protein over-estimates protein in human milk.	• Protein is estimated from the amount of nitrogen in milk. • Nitrogen in human milk can be measured with IR or the Kjeldahl assay. • In human milk, 20–50% of nitrogen is from non-protein sources. • Kjeldahl can be adapted to also measure non-protein nitrogen, whereas IR cannot distinguish non-protein nitrogen and uses a correction factor to estimate true protein value (e.g., Miris IR calculates true protein as [crude protein]*0.8)
	True Protein	Accounts for the non-protein nitrogen (NPN) either through measurement of NPN or applying a constant discount. This is the most nutritionally relevant value.	
Energy	Gross Energy	Energy provided when all organic molecules are burned in a bomb calorimeter.	• Metabolizable energy requires distinguishing protein from non-protein nitrogen, and lactose from HMOs. • FAO’s recommended metabolizable energy conversion factors are 9, 4, 4, and 2 per gram of fat, true protein, digestible carbohydrate (lactose) and indigestible carbohydrate (HMOs). • Energy estimates from IR are based on how the instrument is calibrated and configured. Miris IR instrument estimates gross energy using crude protein, total fat, and total carbohydrate measures.
	Metabolizable Energy	The energy that is biologically available to an infant after digesting and metabolizing nutrients.	

## Protein

Milk proteins are sources of amino acids for the growing infant [1], and also have bioactive functions [2]. Protein concentration declines after delivery, falling sharply in the first month [3], and is higher after preterm birth versus full-term, particularly in the first 4 weeks postpartum [3]. Protein concentrations do not vary by time-of-day [4], or by sample collection method [5], suggesting that there are few limitations in obtaining a representative sample of milk for protein analysis.

A common approach to measuring protein in foods is to assess the nitrogen, since most nitrogen in foods is found in the protein fraction. Measured nitrogen is then used to estimate protein content using a general or food-specific nitrogen conversion factor. Non-protein nitrogen (NPN) in different food varies and can influence the accuracy of nitrogen-based protein quantification techniques. For example, human milk averages 25–30% NPN, with values reported as high as 55% [6, 7], whereas cow’s milk contains ~5% NPN [8]. For more accurate assessment of protein, NPN is accounted for through direct measurement or through estimation. The term “crude protein” (also referred to as “total protein”) assumes that all nitrogen in food is attributed to protein, while the term “true protein” accounts only for the protein fraction. From a clinical standpoint, crude/total protein overestimates protein due to the large fraction of NPN in human milk. In other words, true protein–rather than crude/total protein–is more clinically relevant for nutritional purposes.

The gold standard for measuring nitrogen in milk is the modified Kjeldahl method, a wet-lab method that is practical in food production and research settings. In clinical settings, IR is more practical and quantifies nitrogen based on the infrared radiation absorbed by nitrogen-containing bonds. Importantly, IR cannot distinguish between nitrogen in protein versus NPN, therefore a standard correction factor is usually applied to estimate true protein.

Recent studies have compared the accuracy of different IR instruments. Kwan et al. evaluated two different IR instruments in 13 clinical and milk bank settings and found that protein precision and accuracy varied between sites regardless of the instrument used. Performance improved with appropriate calibration using reference standard materials and/or use of correction factors, as well as use of good laboratory practices [9]. Perrin et al. evaluated four IR instruments used in three milk banks against accuracy thresholds determined by the FDA (±15% for crude protein) and reported that all analyzers typically met this threshold [10]. To achieve accurate true protein estimates, instruments required different correction factors ranging from 20 to 30%, highlighting the importance of instrument monitoring and calibration. When NPN in human milk is high (e.g. 40–50%), true protein values will be overestimated using a standard correction factor in the range of 20–30% (actual percent NPN less standard correction factor).

### Clinical pearls

Given variations in how protein is reported, to better understand nutritional information provided about MOM and PDHM, clinicians should ask: (i) Does this value represent true protein or crude/total protein? Implications: crude/total protein includes NPN and overstates available protein. (ii) If true protein, how was NPN accounted for (measured vs standard correction factor)? Implications: Use of a standard correction factor may overstate true protein by 20–30% in situations where actual milk NPN is higher than the correction factor.

## Carbohydrates

The human milk carbohydrate matrix is influenced by lactation stage and consists of lactose (70–85%), human milk oligosaccharides (HMOs; 15–25%), and monosaccharides (<1%) [1, 11]. Lactose increases significantly in the first weeks postpartum and then remains stable, while many HMOs decline throughout lactation and are highly variable between individuals [12–15]. Lactose provides a major source of energy, while HMOs are indigestible by the infant, though can contribute some energy when metabolized by gut microbiome. Thus, being able to distinguish between lactose and HMOs is critical for reliably estimating available energy values. Carbohydrate concentrations in human milk do not vary by time of day [4], or by sample collection method [5], suggesting that there are few limitations in obtaining a representative milk sample for carbohydrate analysis.

Two methods – one enzymatic, and the other using chromatography – were recently validated for measuring lactose in human milk [16], however, these techniques are typically not feasible to implement in clinical settings. Lactose and HMOs are not distinguishable by IR devices because of similarities in their chemical structures, specifically the terminal lactose unit on each HMO [16, 17]. Carbohydrate estimates using IR are strongly influenced by calibration and instrumentation [9, 10]. A study of 4 different IR analyzers used in 3 milk bank settings reported significantly different results across a shared set of samples (ranging from an average of 5.9–7.9 g/dL) [10]. The instruments were all highly correlated for their carbohydrate readings (correlation coefficient of 0.87–0.98; unpublished data) suggesting they were reading similar signals, but differed in how carbohydrate values were computed. Validation studies report that IR and chemical methods for lactose quantification are not well-correlated [9, 18]; however IR instruments are still able to achieve FDA accuracy standard (±15%) when estimating carbohydrates [10, 19] due to the limited variability of carbohydrates in human milk [12, 20]. Whether IR instruments are calibrated to lactose or to total carbohydrates will strongly influence carbohydrate estimates. Instruments calibrated to total carbohydrates will systematically over-report the available energy in human milk by assigning the same energy values to digestible (lactose) and indigestible (HMOs) carbohydrates [21]. The impact of this will be further discussed in the “Energy” subsection of this paper.

### Clinical pearls

Given variations in how carbohydrates are reported, to fully understand nutritional information provided about MOM and PDHM, clinicians should ask: (i) Do carbohydrate values reflect lactose (digestible carbohydrate), or total carbohydrates (digestible and indigestible carbohydrates)? Implication: if values reflect total carbohydrates, available energy will be overstated.

## Fat

Lipids in human milk are primarily composed of fatty acids esterified to glycerol (i.e. triglycerides, 98%) and smaller quantities of cholesterol and phospholipids [1, 22]. Triglycerides provide 45–50% of the total energy in human milk [20], while other lipids found in the milk fat globule membranes are associated with important health benefits [23]. Fat composition is highly variable, with estimates for mature milk averaging from 3.3 to 3.7 g/dL [3].

Fat composition may be influenced by factors including maternal adiposity and maternal diet [24–26], and changes throughout the day, with highest concentrations reported in the evening [4]. Fat is profoundly influenced by sample collection and handling practices, with post-feed collections being significantly higher than pre-feed collections [5]. Thus, sampling and handling can markedly influence the trustworthiness of values regarding human milk fat [1, 27]. Strategies for collecting a representative sample for clinical decision making include using an aliquot from milk pooled for feeding over 24-hours or having mothers pool their milk expressions over 24-h [1, 28].

The gold standard method for analyzing human milk fat is the gravimetric method which is based on the extraction and weighing of fat [1]. This wet-laboratory-based technique requires proper handling of organic solvents and is not practical clinically. Within the NICUs and milk banks, both creamatocrit and IR analyzers have been employed for rapid measurement of human milk fat.

Creamatocrit involves placing a small volume of milk (about 0.1 mL) in a measurement tube which is centrifuged to separate the milk into fat and aqueous layers. The fat layer is measured and compared to the size of the total column. Creamatocrit percentage is then converted to concentration (g/dL) using a published equation [29]. Creamatocrit is reliable for estimating fat within a typical range, but becomes unreliable when composition is below 2 g/dL, or when milk has undergone prolonged storage, due to hydrolysis of triglycerides into free fatty acids that may pack more tightly [30–32].

Studies to evaluate the reliability of IR for measuring fat in human milk report strong agreement with chemical reference methods when samples are appropriately homogenized [9, 10, 18]. One study reported improved IR accuracy when milk was sonicated using sound waves [27]. Further, a recent study noted that sample volumes of 4.5 mL versus 1.5 mL produced more reliable fat measurements [9], suggesting that clinicians may want to prioritize collecting larger sample volumes when possible.

### Clinical pearls

Standardized sample collection and careful handling techniques are critical for generating reliable information about fat composition of human milk. IR analysis is reliable for measuring fat in human milk when appropriate sample collection and handling processes have been followed. Creamatocrit reliability decreases when fat concentrations are low, and when milk has undergone storage.

## Energy

Gross energy refers to the total energy in foods if all organic constituents were biologically available for fuel. It is typically assessed by burning food in a bomb calorimeter. Energy can also be estimated in foods by applying conversion factors to different nutrient classes. Metabolizable energy differs from gross energy as it reflects the biologically available energy and does not include organic molecules that are not readily converted to energy (e.g. vitamins, NPN). The Food and Agriculture Organization (FAO) recommends reporting the metabolizable energy in foods using the Atwater conversion factors of 9 kcal/g of fat, 4 kcal/g of true protein, 4 kcal/g of digestible carbohydrates, and 2 kcal/g of indigestible carbohydrates (HMOs) [21]. Balance studies of term and preterm infants support the principle that not all energy in human milk is available for the infant [33, 34]. Thus, metabolizable energy values are most relevant when estimating energy in human milk [35].

IR instruments estimate energy in human milk by applying conversion factors to the different macronutrients. Some IR instruments are calibrated and configured to estimate the metabolizable energy using metabolizable nutrients (e.g. true protein, lactose) and the Atwater conversion factors, while others estimate the gross energy using gross nutrients (e.g. crude protein, total carbohydrates) and alternate conversion factors [36]. In a study that compared a variety of energy conversion factors that have been used in the literature to quantify the energy in human milk, the FAO-recommended Atwater factors were consistently 2–3 kcal/oz lower than energy values generated by other conversion factors [37]. A review of preterm milk composition comparing gross energy to metabolizable energy reported 5–10% higher estimates when gross energy was used [35]. Overall, IR instruments that are calibrated and configured to estimate energy using crude/total protein and total carbohydrate values and/or conversion factors other than Atwater will likely overestimate the available energy compared to the more clinically relevant metabolizable energy (based on true protein, lactose, and Atwater conversion factors).

### Clinical pearls

In the absence of standards for how human milk energy values are reported, to better understand energy information provided about MOM and PDHM, clinicians should ask: (i) Was energy measured using bomb calorimetry (gross energy) or estimated using conversion factors? (ii) If estimated, were they based on metabolizable nutrients (e.g lactose and true protein) or gross nutrients (e.g. crude protein, total carbohydrates)? (iii) What conversion factors were used to compute energy? Implications: energy values obtained using crude protein, total carbohydrates, and/or using factors other than the Atwater conversion factors may potentially overstate available energy by up to 10%.

## Important clinical considerations

### When using pasteurized donor human milk

The World Health Organization (WHO) recommends PDHM as a preferential alternative for feeding small vulnerable newborns in the absence of adequate volumes of maternal milk [38]. While many country-specific milk banking guidelines exist for the management and safety of PDHM, quality control efforts have historically focused on microbial contaminants, not nutrient content [39]. Multi-donor pooling and including milk expressed from mothers of preterm infants and milk expressed in early versus later postpartum periods are strategies used by many milk banks to reduce nutrient variability and avoid low protein content [40, 41]. Mitigating nutrient loss during processing, improving pooling protocols, and creating nutrient and labeling standards for PDHM may improve infant growth and potentially the bioactivity of human milk products [42]. In the future, milk banks will need to standardize target goals for fat and protein content so clinicians can confidently adhere to preterm nutrition protocols [43].

#### Clinical pearls

To fully understand the PDHM hospitals are using, clinicians may find it helpful to contact their milk provider and ask questions related to milk pooling practices and nutritional labels including: (i) How many donors are typically pooled together during PDHM production? (ii) Does the milk bank routinely analyze milk for nutrient content? (iii) Does nutritional information reflect true protein or crude protein? (iv) Does nutritional information reflect gross or metabolizable energy? Implications: As noted above, true protein and metabolizable energy are more accurate indicators whereas crude protein and gross energy overestimate nutrients that are available for the infant.

### When fortifying mother’s milk or pasteurized donor human milk

Although not explicitly stated, typical clinical guidelines for human milk fortification rest on a premise that milk composition aligns with published reference values. While practical, this approach does not consider the true variation in human milk composition. Fortification strategies based upon assumed reference values for protein and energy – often referred to as ‘standard” fortification – may lead to under-fortification when the actual milk—whether maternal or donor—is considerably lower than reference values.

Individual human milk analysis in the NICU and/or nutritional analysis and labeling of PDHM products are intriguing strategies to inform targeted fortification with the goal of ensuring that recommended intakes are met. This approach is often referred to as individualized fortification. Emerging literature and ongoing trials are creating an evidence base regarding the extent to which routine clinical milk analysis, or individualized fortification, translates to improved clinical outcomes [44]; an alternative strategy is to incorporate “as needed” clinical milk analysis in the evaluation of infants with growth faltering. Before analyzing milk, we recommend starting by ensuring that inadvertent fat loss is not occurring, for example fat loss during transfer of milk between containers and/or unnecessarily prolonged feeding times with milk fed using a pump [45], and that standard fortification has been maximized according to the local practice guideline. If growth remains inadequate, milk analysis may provide helpful insights, either supporting further fortification, or shifting the focus to other causes of growth impairment such as an infant with increased nutritional requirements, malabsorption, or genetic causes. A potential unintended consequence of nutrient analysis of MOM is that parents begin to view their milk as “inadequate,” so we caution about messaging when performing nutrient analysis on MOM.

#### Clinical pearls

When interpreting clinical milk analysis and/or PDHM labels to inform fortification interventions, it is critical to understand the importance of using the true protein value (e.g., crude/total protein discounted for NPN) and the metabolizable energy values to avoid overestimating protein and energy intakes and thereby under-fortifying. It is also critical to obtain representative milk samples for analysis, to ensure trustworthy results.

## Conclusion

While macronutrient information about MOM and PDHM is increasingly available in clinical settings, there are currently no standards for measuring and reporting this data. Clinicians can ask questions to better understand what nutrient values are being reported, how they were assessed, and how milk banks pool donors, which can help to put nutrient values in context, and create more rigorous research for exploring the impact of human milk feeding on infant outcomes. In settings where clinicians do not have access to labeled or analyzed human milk products, we direct readers to systematic reviews that considered the macronutrient measurement issues described in this report [3, 46]. A more recent review did not consider analytical methods and consistently reported higher protein values by 0.2–0.5 g/dL [47], further highlighting the importance of analytical methods.
